# Solid Harmonic Wavelet Bispectrum for Image Analysis

**DOI:** 10.1002/advs.202517383

**Published:** 2025-12-03

**Authors:** Alex Brown, Mathilda Avirett‐Mackenzie, Carolin Villforth, Georgios Exarchakis

**Affiliations:** ^1^ Department of Computer Science University of Bath Claverton Down Bath BA2 7AY UK; ^2^ Department of Physics University of Bath Claverton Down Bath BA2 7AY UK

**Keywords:** bispectrum, higher‐order features, rotation invariance, solid harmonics

## Abstract

The Solid Harmonic Wavelet Bispectrum in 2D provides a multi‐scale, rotation‐ and translation‐covariant representation that preserves relative phase and captures higher‐order interactions between wavelet responses. This representation encodes rich structural information in a data‐efficient and interpretable form. Applications across texture classification, medical imaging, galaxy merger regression, and image reconstruction demonstrate that phase‐sensitive, cross‐scale interactions enhance discriminative power, model complex dependencies, and retain sufficient information for accurate reconstructions. By embedding roto‐translation invariance and preserving relative phase, the operator captures structural features often lost in conventional scattering methods, enabling robust performance in low‐data regimes. Cross‐scale and higher‐order interactions further enrich the representation, allowing nonlinear dependencies between features to be encoded without learning. Results show competitive or superior performance compared to deep learning models in tasks where symmetries and structural cues dominate, highlighting the potential of phase‐sensitive, symmetry‐aware wavelet representations as a versatile tool for signal and image analysis.

## Graphical abstract

The Solid Harmonic Wavelet Bispectrum (SHWB), a rotation‐ and translation‐invariant descriptor that captures higher‐order (phase) correlations in signals, is introduced. Combining wavelet scattering, bispectral analysis, and group theory, SHWB achieves interpretable, data‐efficient representations and demonstrates competitive performance across texture, medical, and astrophysical datasets, especially in data‐scarce regimes.

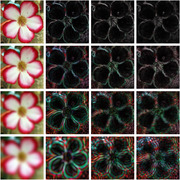

## 1 Introduction

In most scientific experiments, and in data recording more broadly, sensors rarely measure the physical quantities we truly care about. Instead, they capture indirect consequences: light patterns rather than molecular structures, electrical potentials rather than brain states, or pixel arrays rather than 3D scenes. A central task of science has always been to transform these observations into meaningful representations of the underlying phenomena. Machine learning has proven remarkably effective in this regard, learning such transformations directly from examples of data and labels. Yet there is also a complementary path: to design transformations that explicitly encode the symmetries of the physical world. By aligning our representations with these fundamental invariances, we obtain a more principled and geometrically grounded understanding of data—an approach especially powerful in data‐scarce scientific domains, where statistical learning alone may be unreliable.^[^ ^1^, ^2^ ^]^

A well‐defined strategy within this alternative framework that allows rigorous analysis of the methodology is to build representations that are invariant to nuisance transformations like translation and rotation. The standard method involves two steps: first, extracting features that are covariant—that is, the transformation may be applied before or after the feature extraction with consistent effect—and second, applying a pooling operation to achieve invariance. This principle has deep roots in harmonic analysis and has been central to the development of geometric deep learning.^[^ ^3^, ^4^ ^]^ While standard Convolutional Neural Networks (CNNs) can learn approximations of these properties, they do so statistically and require extensive data augmentation to generalize across viewpoints. This motivates the design of architectures with built‐in geometric guarantees.

Wavelet scattering networks offer a compelling implementation of this principle, avoiding learned filters entirely. They construct translation‐invariant representations through a cascade of predefined wavelet transforms, modulus nonlinearities, and spatial averaging. These handcrafted feature extractors have proven highly data‐efficient, matching deep learning performance on tasks from quantum chemistry to texture classification.^[^ ^5^, ^6^, ^7^ ^]^ Extensions using oriented or solid harmonic wavelets have successfully produced rotation‐invariant descriptors. However, a key limitation of these methods is their reliance on second‐order statistics; the complex modulus operation discards phase information that can encode important higher‐order structural dependencies.

Conventional scattering approaches, relying on second‐order correlations, primarily capture global isotropic structure such as average intensity, texture energy, or shading. While valuable, these statistics do not encode geometric arrangement. By contrast, third‐order correlations are sensitive to how features are jointly arranged: whereas pairwise statistics capture global similarity, triplets can discriminate local structure and encode directionality. This makes them far more expressive for describing geometry. Exploring third‐order correlations in the setting of rotationally symmetric operators such as solid harmonic wavelets, is particularly intriguing. Because these operators are covariant to rotations, their bispectra naturally yield a more discriminative set of features while producing orientation‐invariant descriptors.

Building on this motivation, this work focuses on the Solid Harmonic Wavelet Bispectrum (SHWB), a new operator that integrates multi‐scale analysis, higher‐order statistics, and group‐theoretic invariance. Whereas conventional scattering approaches rely primarily on second‐order correlations—discarding potentially informative phase interactions—the SHWB incorporates third‐order statistics through bispectral analysis of solid harmonic wavelet responses. This enables the capture of nonlinear couplings between rotational components across scales, yielding representations that are both theoretically covariant to rotations and translations, and empirically more discriminative. Preliminary experiments suggest that this framework enhances linear separability and offers significant advantages in medical imaging and related fields.

While the technical foundations of our approach draw on signal processing and computer vision, the principles extend naturally to a broad range of scientific domains. Many experimental datasets—from medical imaging to astrophysics—exhibit underlying symmetries and complex multi‐scale structures. By embedding these invariances directly into the representation, the Solid Harmonic Wavelet Bispectrum provides a data‐efficient, interpretable, and a physically grounded alternative to learned models. This positions our work not only within the computational signal processing literature, but also as broadly relevant to researchers seeking principled, symmetry‐aware representations across the sciences.

In what follows, we situate the SHWB within the broader scientific effort to design invariant and interpretable representations, reflecting on how such work illustrates the ongoing interplay between mathematics, computation, and the sciences. As our university marks its 60th anniversary, this line of research exemplifies our commitment to foundational advances that not only deepen theoretical understanding, but also open new avenues for practical impact across disciplines.

## 2 Background

### 2.1 Symmetry‐aware Signal Processing

A common theme in signal processing is to identify structures in the signal that remain unaltered through variations. It was therefore natural to turn to approximations built on periodic functions that analyse signals based on their repetitive structure. As a result, Fourier analysis quickly became the central tool for approaching signal processing problems. When dealing with translations, Fourier components gave rise to translation invariance naturally. More complex invariants, such as rotations, required explicit modeling through moment methods, for example, Hu's classical moment invariants^[^ ^8^ ^]^ and later Zernike moments,^[^ ^9^ ^]^ which reduced variability through global averaging. These methods offered global invariance but not local geometric description.

A significant step in signal processing was the development of multiresolution analysis and wavelet theory,^[^ ^10^ ^]^ enabling the signals to be decomposed across scales with both time and frequency localization. The introduction of steerable and oriented wavelets^[^ ^11^, ^12^ ^]^ added the ability to capture directional information, laying the groundwork for rotation‐aware representations. More recently, influenced by deep learning, wavelet scattering networks^[^ ^5^ ^]^ were developed to provide more sophisticated invariants by introducing a cascade of linear and non‐linear wavelet operators. Because these operators are defined in Fourier space, the modulus nonlinearity discards phase while preserving amplitude, yielding a covariant representation with respect to translations. Subsequent local averaging then produces invariants. The result is a handcrafted, mathematically grounded analogue of a deep network, offering translation invariance and stability guarantees without learned parameters.

Symmetry modeling through scattering networks was extended using steerable^[^ ^13^ ^]^ and solid harmonic wavelets,^[^ ^7^ ^]^ which incorporated orientation and spherical symmetries, enabling roto‐translation invariance. These methods proved remarkably effective in domains from texture discrimination^[^ ^14^ ^]^ to quantum chemistry,^[^ ^7^ ^]^ domains where data is scarce and physical symmetries are critical.

**Figure 1:**
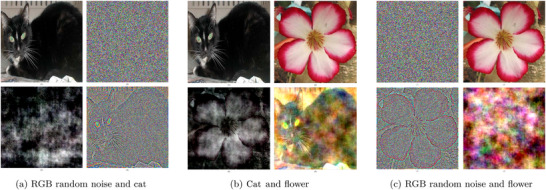
Demonstration of the structure preserved by the phase when taking the Fourier transforms of images and swapping their phase components, inspired by ref. [15]. In each of the figures (a– c), the top row shows two original and independent images. The bottom left takes magnitude from the top left and phase from the top right image. The bottom right image takes magnitude from the top right image and phase from the top left image. Natural images of a child and a flower are sourced from ref. [20]. Plots show that phase carries structural information such as edges, while the magnitude encodes overall signal energy.

Yet a key limitation to all these methods is that the complex modulus discards phase information. Phase encodes the precise alignment of features within a signal and, although highly sensitive to variation, it carries much of the perceptual structure. **Figure** **1** reproduces the classic experiment of Oppenheim and Lim,^[^ ^15^ ^]^ which demonstrates that retaining Fourier phase preserves image structure, whereas retaining only Fourier magnitudes does not. Because phase features are unstable, however, most signal representations have traditionally avoided them, relying instead on invariant magnitude‐based statistics. Subsequent work in higher‐order spectral analysis^[^ ^16^, ^17^ ^]^ showed that invariants can be built from the relative phase between frequency components. The bispectrum, as a third‐order statistic, makes this explicit by capturing phase couplings among triplets of frequencies, which encode geometric dependencies lost in second‐order correlations. Importantly, the bispectrum vanishes for Gaussian processes, so any non‐zero bispectral structure reveals higher‐order dependencies. While redundancy in wavelet design can, in principle, allow approximate phase reconstruction,^[^ ^18^ ^]^ scattering frameworks do not explicitly capture such higher‐order relationships. Extensions such as phase harmonic correlations^[^ ^19^ ^]^ attempt to reintroduce them, but only under constrained signal classes.

The study of higher‐order spectra (HOS) predates wavelet scattering transforms and originates in non‐Gaussian signal analysis.^[^ ^16^ ^]^ The bispectrum is a third‐order statistic that captures interactions among triplets of frequencies and preserves information about phase relationships the power spectrum discards. Due to the significance of phase information, we find applications of the bispectrum across fields as diverse as oceanography, speech processing, and plasma physics. Wavelet‐domain extensions introduced multi‐scale bispectral analysis,^[^ ^21^, ^22^, ^23^ ^]^ and more recent work proposed normalized “bispectral densities” for greater interpretability.^[^ ^24^ ^]^ However, these approaches remain rotation‐sensitive and lack the geometric guarantees of scattering.

From a geometric perspective, bispectral methods on spherical domains have also been developed. For example, Kakarala^[^ ^17^ ^]^ projected signals onto solid harmonics before applying the Fourier bispectrum, while Kondor^[^ ^3^ ^]^ constructed rotationally invariant bispectra using Clebsch–Gordan coefficients. While elegant, these methods often require spherical projections that distort data or involve redundant computations, limiting scalability.

In parallel, the field of geometric deep learning has pursued symmetry‐preserving architectures through learning. Group‐equivariant CNNs (G‐CNNs)^[^ ^4^ ^]^ extend convolution and pooling to enforce equivariance to specified transformation groups, while harmonic networks (H‐Nets)^[^ ^25^ ^]^ employ complex circular harmonics to achieve continuous rotational equivariance. More recent work on bispectral neural networks^[^ ^26^ ^]^ takes this further, learning invariants directly by optimizing orbit separation losses in bispectral space. While powerful, these approaches require substantial training data, assume a known symmetry group, and incur computational costs that limit interpretability and scalability.

The work discussed so far in this section offers several complementary strengths and weaknesses. Wavelet scattering provides multi‐resolution representations with stability guarantees and data efficiency, but it discards higher‐order dependencies by removing phase. Higher‐order spectra recover this missing structure by encoding nonlinear phase interactions, although they are usually applied without an explicit link to geometric symmetries. Geometric deep learning instead builds invariances directly into trainable architectures, offering remarkable flexibility, but at the cost of heavy data requirements and limited interpretability.

The Solid Harmonic Wavelet Bispectrum (SHWB) is motivated by the idea of combining these perspectives. By replacing the modulus operation in scattering with a bispectral operator, it brings together the stability of wavelets, the structural richness of higher‐order correlations, and the group‐theoretic guarantees of solid harmonics. This synthesis yields a representation that remains interpretable, incorporates rotational and translational invariance by construction, and captures more discriminative geometric features than second‐order methods alone. Related approaches in physics‐informed modeling also explore embedding structured constraints directly into representations to improve interpretability and data efficiency.^[^ ^27^, ^28^ ^]^

In summary, wavelet scattering networks provide stability and data efficiency, higher‐order spectra recover nonlinear phase interactions, and geometric deep learning incorporates explicit symmetry priors. The SHWB unites these strengths, maintaining interpretability while encoding multi‐scale, higher‐order correlations with guaranteed rotational and translational invariance. This makes SHWB particularly suited for scientific datasets where structural information is critical and training data is limited. In the following sections, we demonstrate its advantages on texture, medical, and astrophysical benchmarks.

### 2.2 Solid Harmonic Wavelet Transform

The Solid Harmonic Wavelet Transform^[^ ^7^ ^]^ offers rich roto‐translation invariants for 2D and 3D signals. Solid harmonics are solutions to the Laplace equation in polar (2D) or spherical (3D) coordinates and form a complete, orthogonal basis on the unit circle or sphere. Any square‐integrable function on these domains can be expressed as a weighted sum of solid harmonic components.

To achieve spatial localization, these basis functions are modulated by a Gaussian envelope, forming solid harmonic wavelets.^[^ ^7^ ^]^ They are zero‐mean, rapidly decaying, and typically normalized to unit energy. In two dimensions, a solid harmonic wavelet is parameterized by an angular frequency ℓ and scale *j*, and is defined as:

(1) $$\psi_{j,\ell }\left(r,\varphi \right)=\frac{1}{\sqrt{{\left(2\pi \sigma_{j}^{2}\right)}^{2}}}e^{-\frac{r^{2}}{2\sigma_{j}^{2}}}\;r^{\ell }e^{i\ell \varphi }$$

where the standard deviation σ_*j*_ = 2^*j*^σ_0_ grows dyadically with *j*. To work with convolutions, it is often simple to represent the wavelets in Cartesian coordinates *u* = (*r*cos φ, *r*sin φ)^*T*^ as ψ_*j*, ℓ_(*u*).

We obtain a filter bank by varying ℓ and *j* that captures structure across different scales and angular frequencies. These wavelets are zero‐mean, band‐pass, and decay rapidly away from the origin, making them suitable for multiscale signal analysis.^[^ ^29^ ^]^ When ℓ = 0, the wavelet reduces to an isotropic Gaussian, which acts as a low‐pass filter rather than a true admissible wavelet. Together, the low‐pass and band‐pass filters form a complete multiscale representation of the signal.

Given a signal $x:R^{2}\to C$, the solid harmonic wavelet transform is the Cartesian convolution:

(2) $$W\left[x,j,\ell \right]\left(u\right)=\left(x★\psi_{j,\ell }\right)\left(u\right)=\int_{R^{2}}x\left(v\right)\;\psi_{j,\ell }\left(u-v\right)\;dv$$

which yields localized responses indexed by scale *j*, angular frequency ℓ, and location *u*.

We denote a rotation *R*_θ_ as *R*_θ_ *x*(*u*) = *x*(*R*_−θ_ *u*), or in polar coordinates *R*_θ_ *x*(*r*, φ) = *x*(*r*, φ − θ). A rotated version of the solid harmonic wavelet in Equation (1) satisfies:

(3) $$\begin{array}{cc} R_{\theta }\psi_{j,\ell }\left(r,\varphi \right) & =\psi_{j,\ell }\left(r,\varphi -\theta \right) \end{array}$$

(4) $$\begin{array}{cc} & =g_{j}\left(r\right)\;r^{\ell }e^{i\ell (\varphi -\theta )} \end{array}$$

(5) $$\begin{array}{cc} & =e^{-i\ell \theta }\;\psi_{j,\ell }\left(r,\varphi \right) \end{array}$$

therefore solid harmonic wavelets are *steerable*: any rotated wavelet is proportional to the original wavelet via a simple phase factor.

For the convolution of a wavelet with a rotated signal, we have:

(6) $$\begin{array}{cc} W\left[R_{\theta }x,j,\ell \right]\left(u\right) & =\int x\left(R_{-\theta }v\right)\;\psi_{j,\ell }\left(u-v\right)\;dv \end{array}$$

(7) $$\begin{array}{cc} & \overset{\;w=R_{-\theta }v\;}{\to }\int x\left(w\right)\;\psi_{j,\ell }\left(u-R_{\theta }w\right)\;dw \end{array}$$

(8) $$\begin{array}{cc} & =\int x\left(w\right)\;\psi_{j,\ell }\left(R_{\theta }\left(R_{-\theta }u-w\right)\right)\;dw \end{array}$$

(9) $$\begin{array}{cc} & \overset{\left(5\right)}{=}e^{i\ell \theta }\int x\left(w\right)\;\psi_{j,\ell }\left(R_{-\theta }u-w\right)\;dw \end{array}$$

(10) $$\begin{array}{cc} & =e^{i\ell \theta }\;W\left[x,j,\ell \right]\left(R_{-\theta }u\right)=e^{i\ell \theta }\;R_{\theta }W\left[x,j,\ell \right]\left(u\right) \end{array}$$

A rotation of the input produces a global phase factor that depends on ℓ and a spatial rotation by θ.

**Figure 2:**
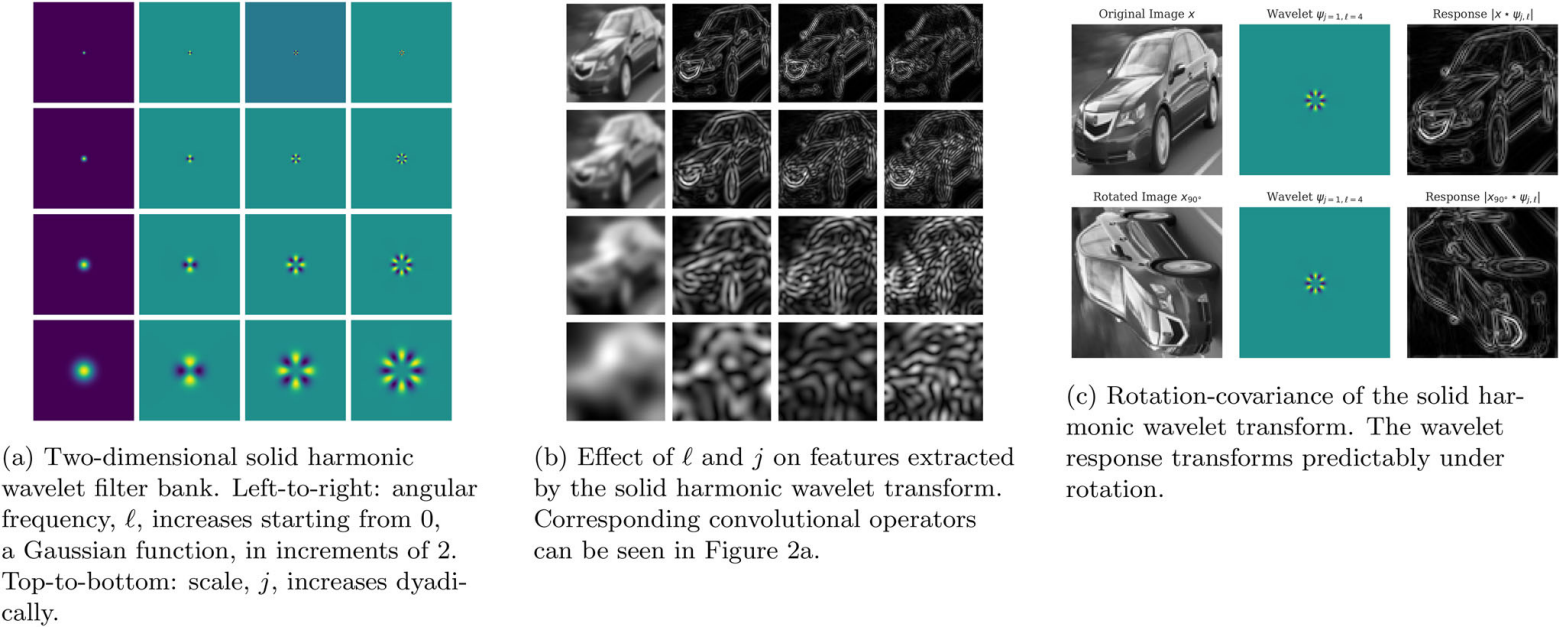
Visualizations of solid harmonic wavelet filters, their wavelet transform responses, and the rotation‐covariance property.

Applying the complex modulus removes the phase in (10),

(11) $$U\left[x,j,\ell \right]\left(u\right)=|W\left[x,j,\ell \right]\left(u\right)|\;\Rightarrow \;U\left[R_{\theta }x,j,\ell \right]\left(u\right)=R_{\theta }U\left[x,j,\ell \right]\left(u\right),$$

so *U* is covariant to rotations: a rotation of the signal is a rotation of the non‐linear wavelet response. The covariant operator yields a different feature for every different pair of ℓ and *j*, spanning different angular oscillations and scales, see **Figure** **2**. From a covariant, we can obtain an invariant feature by integrating over the spatial domain,

(12) $$S_{1}\left[x,j,\ell \right]=\int_{R^{2}}U\left[x,j,\ell \right]\left(u\right)\;du$$

This operation removes the original coordinate system of the signal, which is now represented by the different features spanned by ℓ and *j*. Convolutional operators are by definition translation covariant, and therefore the integration over the spatial component yields translation invariance as well.

#### 2.2.1 Extension to 3D

In three dimensions,^[^ ^7^ ^]^ the angular part of solid harmonics is described by spherical harmonics $Y_{\ell }^{m}$, where ℓ ⩾ 0 is the degree and *m* ∈ {− ℓ, …, ℓ} the order. Under a rotation, the set of harmonics ${\left\{Y_{\ell }^{m}\right\}}_{m}$ of fixed ℓ transforms among itself, preserving the total energy in the ℓ‐th subspace. Therefore, a rotation‐covariant feature can be obtained by computing the squared magnitude summed over *m*:

(13) $$U_{\ell }\left[x,j\right]\left(u\right)=\sqrt{\sum_{m=-\ell }^{\ell }{\left|W\left[x,j,\ell ,m\right]\left(u\right)\right|}^{2}}$$

This quantity is covariant to rotations: rotating the input rotates the spatial pattern of *U*_ℓ_[*x*, *j*] without changing its magnitude.

### 2.3 Solid Harmonic Wavelet Scattering

While solid harmonic wavelets provide multi‐scale, rotation‐ and translation‐covariant features, they capture only first‐order interactions between the signal and the wavelets. To encode more complex relationships between features, one can cascade linear and non‐linear operations, in a manner analogous to deep neural networks. This is the idea behind the *Solid Harmonic Wavelet Scattering* (SHWS) transform.^[^ ^7^ ^]^

#### 2.3.1 Cascaded Transform

Let *U*[*x*, *j*_1_, ℓ_1_] denote the modulus of the wavelet transform at scale *j*_1_ and angular frequency ℓ_1_:

(14) $$U\left[x,j_{1},\ell_{1}\right]\left(u\right)=|W\left[x,j_{1},\ell_{1}\right]\left(u\right)|$$

The first‐order scattering coefficients are obtained by integrating these covariant features:

(15) $$S_{1}\left[x,j_{1},\ell_{1}\right]=\int_{R^{2}}U\left[x,j_{1},\ell_{1}\right]\left(u\right)\;du$$

yielding features invariant to global translations and rotations.

To capture higher‐order interactions, one recursively applies wavelet transforms to the modulus responses:

(16) $$U_{2}\left[x,j_{1},j_{2},\ell_{1},\ell_{2}\right]\left(u\right)=|U\left[x,j_{1},\ell_{1}\right]★\psi_{j_{2},\ell_{2}}\left(u\right)|$$

and integrates to form second‐order scattering coefficients:

(17) $$S_{2}\left[x,j_{1},j_{2},\ell_{1},\ell_{2}\right]=\int_{R^{2}}U_{2}\left[x,j_{1},j_{2},\ell_{1},\ell_{2}\right]\left(u\right)\;du$$

Higher‐order coefficients can be defined similarly by iterating the cascade.

The cascaded SHWS transform introduces a hierarchy of non‐linear interactions between features at different scales and orientations, effectively encoding higher‐order statistical dependencies. In this sense, it provides a computationally efficient surrogate for explicit high‐order spectra like the bispectrum.

Computing the bispectrum directly in Cartesian frequency space is costly, as it requires evaluating all pairs of horizontal and vertical frequencies. By contrast, the angular‐frequency representation of solid harmonic wavelets is intrinsically rotation‐covariant. Each angular frequency ℓ captures correlations across all orientations, so far fewer values of ℓ are needed to cover the same symmetry class. Consequently, calculating the bispectrum over angular frequencies is tractable, and the SHWS transform naturally motivates this approach as an efficient, structured way to encode higher‐order interactions.

### 2.4 Higher‐Order Spectra: The Bispectrum

Second‐order statistics, such as the power spectrum, describe the distribution of signal energy across frequencies. However, they discard phase information, which encodes the relationships between different frequency components. To capture these interactions, one can turn to *higher‐order spectra*, which generalize the power spectrum to third‐ and higher‐order correlations.^[^ ^30^ ^]^

The *bispectrum* is a third‐order statistic defined for a signal *x* as:

(18) $$B\left(f_{1},f_{2}\right)=E\left[\hat{x}\left(f_{1}\right)\;\hat{x}\left(f_{2}\right)\;\bar{\hat{x}\left(f_{1}+f_{2}\right)}\right]$$

where $\hat{x}\left(f\right)$ is the Fourier transform of *x* at frequency *f*, and the overline denotes complex conjugation. Intuitively, the bispectrum measures interactions among frequency triplets (*f*_1_, *f*_2_, *f*_1_ + *f*_2_) and preserves phase relationships that the power spectrum alone cannot.

Because it captures correlations between pairs of frequencies and their sum, the bispectrum is sensitive to nonlinear dependencies in the signal and can reveal quadratic phase coupling. This property makes it particularly useful in contexts where phase encodes important structural or dynamical information, such as in image analysis,^[^ ^31^ ^]^ speech processing,^[^ ^32^ ^]^ and plasma physics.^[^ ^33^ ^]^

A normalized variant, called the *bicoherence*, is often used to isolate phase coupling from overall signal amplitude:

(19) $$\text{Bic}\left(f_{1},f_{2}\right)=\frac{\left|B(f_{1},f_{2})\right|}{\sqrt{E[|\hat{x}\left(f_{1}\right)\hat{x}\left(f_{2}\right){|}^{2}]\;E[|\hat{x}\left(f_{1}+f_{2}\right){|}^{2}]}}$$

which yields values between 0 and 1, where 1 indicates strong phase coupling between the frequency components.

## 3 Methods

### 3.1 Bispectrum of Solid Harmonics

We extend the classical bispectrum framework to the domain of solid harmonic wavelets, combining higher‐order statistics with the localised, geometric structure of wavelet representations. While the scattering transform discards phase information through the complex modulus operator, these operators preserve phase across angular oscillations of different frequencies, retaining nonlinear interactions known to capture structural content.

### 3.2 Solid Harmonic Wavelet Bispectrum

For a given signal $x:R^{2}\to C$, let

(20) $$W\left[x,j,\ell \right]\left(u\right)=\left(x★\psi_{j,\ell }\right)\left(u\right)$$

denote the solid harmonic wavelet transform at scale *j* and angular frequency ℓ. In Fourier space, we define the Solid Harmonic Wavelet Bispectrum at scale *j* over angular frequencies ℓ_1_, ℓ_2_, ℓ_3_ ∈ *L* where ℓ_3_ = ℓ_1_ + ℓ_2_ as:

(21) $$\text{SHWB}\left[x,j,\ell_{1},\ell_{2}\right]=\hat{W}\left[x,j,\ell_{1}\right]\cdot \hat{W}\left[x,j,\ell_{2}\right]\cdot \bar{\hat{W}\left[x,j,\ell_{3}\right]}$$

where $\hat{W}\left[x,j,\ell \right]$ denotes the Fourier transform of the wavelet transform response at scale *j* and angular frequency ℓ. Thus, the Solid Harmonic Wavelet Bispectrum instead measures interactions between wavelet responses of **angular frequency triplets**. While we have demonstrated that increasing angular frequency extracts increasingly granular directional patterns (e.g. textures, see Figure 2), this can abstractly be viewed as an interaction between feature types. To normalize amplitude and isolate phase alignment, we define the bicoherence as:

(22) $$\text{SHWBic}\left[x,j,\ell_{1},\ell_{2}\right]=\frac{\text{SHWB}[x,j,\ell_{1},\ell_{2}]}{∥\hat{W}\left[x,j,\ell_{1}\right]∥\cdot ∥\hat{W}\left[x,j,\ell_{2}\right]∥\cdot ∥\bar{\hat{W}\left[x,j,\ell_{3}\right]}∥}$$

where the Solid Harmonic Wavelet Bicoherence instead measures relative phase coupling between wavelet transform responses, and is bound to [0, 1].

#### 3.2.1 Phase Preservation

Unlike the wavelet scattering transform, which applied a complex modulus to discard the Fourier phase, the Solid Harmonic Wavelet Bispectrum retains the phase component. This allows it to measure higher‐order, quadratic phase couplings that characterize nonlinear interactions and encode key structural information – forming a richer structural representation than wavelet responses alone.

#### 3.2.2 Roto‐Translation Invariants

Since solid harmonics transform predictably under rotation, we can leverage this property in the Solid Harmonic Wavelet Bispectrum. It has been shown that rotations of solid harmonic wavelets satisfy:

(23) $$W\left[R_{\theta }x,j,\ell \right]=e^{i\ell \theta }\;R_{\theta }W\left[x,j,\ell \right]$$

for a spatial rotation *R*_θ_. Rotating a signal has the same effect on its discrete Fourier transform. When substituted into the SHWB definition (Equation 21), and constraining ℓ_3_ = ℓ_1_ + ℓ_2_, the complex phase components therefore cancel:

(24) $$\begin{array}{cc} & \text{SHWB}\left[R_{\theta }x,j,\ell_{1},\ell_{2}\right]={\hat{W}}_{j,\ell_{1}}\left(R_{\theta }x\right)\cdot {\hat{W}}_{j,\ell_{2}}\left(R_{\theta }x\right)\cdot \bar{{\hat{W}}_{j,\ell_{1}+\ell_{2}}\left(R_{\theta }x\right)}\\ & =e^{i\ell_{1}\theta }R_{\theta }{\hat{W}}_{j,\ell_{1}}\left(x\right)\cdot e^{i\ell_{2}\theta }R_{\theta }{\hat{W}}_{j,\ell_{2}}\left(x\right)\cdot \bar{e^{i(\ell_{1}+\ell_{2})\theta }R_{\theta }{\hat{W}}_{j,\ell_{1}+\ell_{2}}\left(x\right)}\\ & =e^{i(\ell_{1}+\ell_{2}-\left(\ell_{1}+\ell_{2}\right))\theta }R_{\theta }\left({\hat{W}}_{j,\ell_{1}}\left(x\right)\cdot {\hat{W}}_{j,\ell_{2}}\left(x\right)\cdot \bar{{\hat{W}}_{j,\ell_{1}+\ell_{2}}\left(x\right)}\right)\\ & =R_{\theta }\text{SHWB}\left[x,j,\ell_{1},\ell_{2}\right] \end{array}$$

i.e. rotation covariance. The SHWB is stable under rotation: a rotated image will produce the same bispectral coefficients multiplied by the spatial rotation. The underlying wavelet convolutions are known to be translation‐covariant,^[^ ^29^ ^]^ meaning the SHWB inherits translation‐covariance. By integrating over the spatial domain, we can reduce these to identical representations – rotation‐ and translation‐invariants. That is, we abstract such transformations out of the representations, reducing variability that does not yield discriminability for our applications. To do so, we extract features using global *L*_*p*_ pooling, defined as the *L*_*p*_ norm of the bispectral coefficients:

(25) $$\text{SHWB}\left[x,j,\ell_{1},\ell_{2}\right]={\left(\int_{R^{2}}{\left|\text{SHWB}\left[x,j,\ell_{1},\ell_{2}\right]\left(\xi \right)\right|}^{p}\;d\xi \right)}^{1/p}$$

The same formulation applies to bicoherence features, too. By applying *L*_*p*_ pooling as a reduction operator, we obtain identical representations for images undergoing rotations and translations. We select *p* to maximize variability for a given task, enhancing discriminability.

#### 3.2.3 Multi‐Scale

Since we dyadically scale solid harmonic wavelets over scales *j* ∈ [0, *J*], we therefore obtain bispectral coefficients at each scale. Consequently, the SHWB captures nonlinear interactions between features at different levels of spatial localization – a clear distinction to classical bispectral analysis, enabled by the utilization of wavelets.

### 3.3 Solid Harmonic Wavelet Scattering Bispectrum

We extend the SHWB to the Solid Harmonic Wavelet Scattering Bispectrum (SHWSB), which introduces inter‐scale interactions to the higher‐order, phase‐sensitive correlations captured by the bispectrum. It remains roto‐translation covariant, which we reduce to an invariant, while cross‐scale dependencies capture structured interactions occurring over multiple scales. Let the first‐ and second‐order linear scattering responses be:

(26) $$\begin{array}{cc} W_{1}\left[x,j_{1},\ell \right] & =x★\psi_{j_{1},\ell } \end{array}$$

(27) $$\begin{array}{cc} W_{2}\left[x,j_{1},j_{2},\ell \right] & =|x★\psi_{j_{1},\ell }|★\psi_{j_{2},\ell } \end{array}$$

where we no longer apply the complex modulus operator to each order, as in Solid Harmonic Wavelet Scattering. Now, we define the bispectral coefficients over intermediate scattering responses in Fourier space for ℓ_1_, ℓ_2_, ℓ_3_ ∈ *L* where ℓ_3_ = ℓ_1_ + ℓ_2_:

(28) $$\begin{array}{cc} {\text{SHWSB}}_{1}\left[x,j_{1},\ell_{1},\ell_{2}\right] & ={\hat{W}}_{1}\left[x,j_{1},\ell_{1}\right]\cdot {\hat{W}}_{1}\left[x,j_{1},\ell_{2}\right]\cdot \bar{{\hat{W}}_{1}\left[x,j_{1},\ell_{3}\right]} \end{array}$$

(29) $$\begin{array}{cc} & {\text{SHWSB}}_{2}\left[x,j_{1},j_{2},\ell_{1},\ell_{2}\right]\\ & \;={\hat{W}}_{2}\left[x,j_{1},j_{2},\ell_{1}\right]\cdot {\hat{W}}_{2}\left[x,j_{1},j_{2},\ell_{2}\right]\cdot \bar{{\hat{W}}_{2}\left[x,j_{1},j_{2},\ell_{3}\right]} \end{array}$$

In this approach, the bispectrum is a measure of interactions between complex‐valued scattering coefficients. We can again normalize by the magnitude of scattering coefficients to quantify relative phase couplings in the bicoherence:

(30) $$\begin{array}{cc} & {\text{SHWSBic}}_{1}\left[x,j_{1},\ell_{1},\ell_{2}\right]\\ & \;=\frac{{\text{SHWSB}}_{1}\left[x,j_{1},\ell_{1},\ell_{2}\right]}{∥{\hat{W}}_{1}\left[x,j_{1},\ell_{1}\right]∥\cdot ∥{\hat{W}}_{1}\left[x,j_{1},\ell_{2}\right]∥\cdot ∥{\hat{W}}_{1}\left[x,j_{1},\ell_{3}\right]∥} \end{array}$$

(31) $$\begin{array}{cc} & {\text{SHWSBic}}_{2}\left[x,j_{1},j_{2},\ell_{1},\ell_{2}\right]\\ & \;=\frac{{\text{SHWSB}}_{2}\left[x,j_{1},j_{2},\ell_{1},\ell_{2}\right]}{∥{\hat{W}}_{2}\left[x,j_{1},j_{2},\ell_{1}\right]∥\cdot ∥{\hat{W}}_{2}\left[x,j_{1},j_{2},\ell_{2}\right]∥\cdot ∥\bar{{\hat{W}}_{2}\left[x,j_{1},j_{2},\ell_{3}\right]}∥} \end{array}$$

#### 3.3.1 Phase Preservation

As with the Solid Harmonic Wavelet Bispectrum, the scattering extension also retains Fourier phase interactions in both first‐ and second‐ order terms. Thus, it captures higher‐order correlations lost in typical scattering networks which apply modulus operators. Our intermediate second‐order responses, *W*_2_[*x*, *j*_1_, *j*_2_, ℓ], retain the modulus nonlinearity over the first convolution to preserve stability of scattering coefficients – increased robustness that offsets the loss of inter‐scale phase. Further, phase interactions within the same orders are retained through the bispectral term.

#### 3.3.2 Roto‐Translation Invariants

First‐order Solid Harmonic Wavelet Scattering Bispectrum features are equivalent to SHWB – the third‐order correlation over wavelet responses in the Fourier domain. Therefore, they too are roto‐translation invariant. Demonstrating this property for second‐order coefficients is slightly more involved. Let us rewrite second‐order linear scattering responses:

(32) $$\begin{array}{cc} W_{2}\left[x,j_{1},j_{2},\ell \right] & =|x★\psi_{j_{1},\ell }|★\psi_{j_{2},\ell }\\ & =|W_{1}\left[x,j_{1},\ell \right]|★\psi_{j_{2},\ell } \end{array}$$

It follows that for a spatial rotation of θ,

(33) $$W_{1}\left[R_{\theta }x,j,\ell \right]=e^{i\ell \theta }\;R_{\theta }W_{1}\left[x,j,\ell \right]$$

which when substituted into Equation (32) cancels the phase factor, as in Solid Harmonic Wavelet Scattering:

(34) $$\begin{array}{cc} W_{2}\left[R_{\theta }x,j_{1},j_{2},\ell \right] & =|e^{i\ell \theta }\;W_{1}\left[R_{\theta }x,j_{1},\ell \right]|★\psi_{j_{2},\ell }\\ & =R_{\theta }W_{1}\left[x,j_{1},\ell \right]★\psi_{j_{2},\ell } \end{array}$$

Rotations of solid harmonics commute, meaning that the second convolution preserves rotation structure and reintroduces the complex phase factor:

(35) $$\begin{array}{cc} W_{2}\left[R_{\theta }x,j_{1},j_{2},\ell \right] & =R_{\theta }W_{1}\left[x,j_{1},\ell \right]★\psi_{j_{2},\ell }\\ & =e^{i\ell \theta }\;R_{\theta }W_{2}\left[x,j_{1},j_{2},\ell \right] \end{array}$$

Therefore, the same property as in the Solid Harmonic Wavelet Bispectrum establishes that for ℓ_1_, ℓ_2_, ℓ_3_ ∈ *L* where ℓ_1_ + ℓ_2_ = ℓ_3_, the phase factor will cancel. This means that both first‐ and second‐ order Solid Harmonic Wavelet Scattering Bispectrum coefficients are rotation covariant. *L*_*p*_ pooling converts them to invariants.

#### 3.3.3 Multi‐ and Inter‐ Scale

A key difference between SHWB and SHWSB is that the scattering bispectrum enriches the representation with cross‐scale dependencies, making it well suited to domains where structures exist across scales. Furthermore, since first‐order coefficients are fundamentally the SHWB, it retains higher‐order interactions between localised features, too. However, it is worth highlighting that in data‐scarce domains, the introduction of large feature counts can make it more difficult for a simple model to learn a complex decision boundary.

### 3.4 Computation of the Bispectrum

Computing the bispectral coefficients efficiently requires careful consideration of the combinatorial growth in angular frequency triplets (ℓ_1_, ℓ_2_, ℓ_3_) that satisfy ℓ_1_ + ℓ_2_ = ℓ_3_. The following implementation strategies help reduce redundant computations and improve performance.

#### 3.4.1 Precomputation

Wavelet responses *W*[*x*, *j*, ℓ] and modulus responses *U*[*x*, *j*, ℓ] are reused across multiple bispectral terms, so they are computed once and cached. For high‐resolution images, responses are processed in batches to control memory usage.

#### 3.4.2 Symmetry

The bispectrum is symmetric in the first two frequencies: ${\text{SHWB}}_{\ell_{1},\ell_{2}}={\text{SHWB}}_{\ell_{2},\ell_{1}}$. Only unique terms with ℓ_1_ ⩽ ℓ_2_ are computed, and triplets violating ℓ_1_ + ℓ_2_ = ℓ_3_ are skipped.

#### 3.4.3 Acceleration

We implement our bispectral operators in PyTorch to leverage GPU acceleration. Convolutions are performed with the Fast Fourier Transform (FFT), extending the Kymatio framework.^[^ ^34^ ^]^

## 4 Results

### 4.1 Texture Classification

For texture analysis, preserving phase information was critical. Human perception of texture similarity has been shown to be dominated by information in Fourier phase, as opposed to magnitude.^[^ ^35^ ^]^ This indicates that in approaching texture analysis, one must prioritize phase preservation, which was known to encode key structural information. To this extent, the solid harmonic wavelet bispectrum was benchmarked against prior scattering techniques on an established texture classification benchmark: KTH‐TIPS.^[^ ^36^ ^]^ The goal was not to set state‐of‐the‐art results, but to demonstrate that phase‐sensitive, higher‐order interactions were crucial in tasks where variability was driven by structural properties – interactions that the solid harmonic wavelet bispectrum encodes.

KTH‐TIPS is comprised of 10 classes, each with 81 samples subject to variations in scale, shear and illumination. Linear SVM classifiers were trained over 200 train‐test splits and varying training set sizes, consistent with.^[^ ^13^ ^]^ For the solid harmonic transform, ℓ = 5, *J* = 3were used, and estimated the integrals using *L*_*p*_ norms for *p* ∈ {0.5, 1, 2}. This controlled experiment offers an assessment of the statistical robustness of the operator, and it's ability to encode structural variability under limited data. Moreover, the side‐by‐side comparison with other wavelet‐based techniques provides meaningful insight into the strengths of phase‐sensitive methods. Specifically, comparisons were made to locally translation‐invariant, roto‐translation invariants and an “enhanced” roto‐translation invariant, which employs further logarithmic nonlinearities, averaging over scales and scale augmentations. The enhanced version was included for completeness, citing it was therefore not a direct comparison to the approaches. The linear SVM was known to hold similar properties to the PCA classifier employed in ref. [13], ensuring a fair comparison.^[^ ^37^ ^]^ demonstrates marginal improvements with the PCA classifier, suggesting that results with the representations could further be improved.

**Table** **1** reports classification performance across different training set sizes. Results reveal a consistent advantage of bispectral interactions, especially in the lowest data regimes. With as few as five training samples, the operators instantaneously yield highly discriminative features for texture classification, with a marked improvement over classical scattering operators. This highlights that phase‐sensitive higher‐order statistics enhance representational expressivity in precisely the setting where phase variability was critical. Moreover, the nonlinear interactions between features at each scale provide a richer representation that extends beyond the information contained in individual wavelet responses.

**Table 1:** Mean and standard deviation accuracy over 200 random training subsets of increasing sizes on KTH‐TIPS.

Features	5 samples	20 samples	40 samples
Translation scattering^[^ ^13^, ^37^ ^]^	69.1 ± 3.5	94.8 ± 1.3	98.0 ± 0.8
Roto‐translation scattering^[^ ^13^ ^]^	69.5 ± 3.6	94.9 ± 1.4	98.3 ± 0.9
SHWS	86.9 ± 0.8	96.1 ± 0.3	98.5 ± 0.4
SHWB	87.7 ± 1.7	94.8 ± 0.5	96.9 ± 0.3
SHWBic	87.2 ± 1.3	96.5 ± 0.6	99.3 ± 0.3
SHWSB	88.6 ± 2.5	95.1 ± 0.3	97.4 ± 0.3
SHWSBic	85.1 ± 0.9	97.0 ± 0.7	98.6 ± 0.2
Enhanced roto‐translation scattering ^[^ ^13^ ^]^	84.3 ± 3.1	98.3 ± 0.9	99.4 ± 0.4

A notable distinction emerges between bispectrum and bicoherence variants. In lower data regimes, the bispectrum yields stronger performance, suggesting higher‐order energy couplings introduce meaningful illumination‐driven variability. However, as the training set grows, bicoherence improves, reflecting its ability to encode stable relative phase interactions that become increasingly informative with sufficient data. Explicitly modeling coupling between wavelet responses captures discriminative structure beyond energy statistics alone.

Finally, higher‐order scattering coefficients which encode cross‐scale interactions yield minor improvements in this task, suggesting that the multi‐scale bispectral interactions encode much of the meaningful variability. While higher‐order scattering enriches the representation incrementally in the lower data regimes, the explicit phase‐sensitive couplings were essential for robust discrimination, and yield the most significant improvements. For example, in the largest training set, the solid harmonic wavelet bicoherence outperforms the scattering bicoherence.

Together, these findings support a central belief in this work: phase‐sensitive higher‐order interactions provide richer, more stable features than traditional energy‐based scattering in tasks where structural information is important.

### 4.2 Medical Imaging

Deep learning approaches to diagnostic tasks in medical imaging are often limited by the availability of centralized, large‐scale datasets.^[^ ^38^ ^]^ In many clinical settings, annotated data is scarce, making it difficult for learned filters to generalize reliably. In contrast, symmetry‐aware wavelet‐based approaches embed key invariances structurally, such as translation and rotation, which abstract the need to generalize across nuisance transformations. This yields meaningful features without requiring large‐scale optimization, leading to increased data efficiency and strong performance on such tasks, where data is scarce.

These capabilities were demonstrated over the MedMNIST dataset collection,^[^ ^39^, ^40^ ^]^ a standardized set of challenges with varying demands on structural sensitivity:

**BreastMNIST (780 samples)** is a binary classification task to diagnose breast tumors, where global symmetries are strong signals for inference. Malignant tumors are irregular, while benign were smooth.

**RetinaMNIST (1600 samples)** is an ordinal regression to grade the severity of diabetic retinopathy, requiring the modeling of localized anomalies such as hemorrhages, exudates and microaneurysms.

**DermaMNIST (10 015 samples)** is the seven‐class classification of pigmented skin lesions, which relies on complex multiscale textures. Classes are discriminable based on indicators such as asymmetry, border regularity and color distribution.

To evaluate, a single linear layer with *L*_2_ regularization and singular value decomposition was selectively employed. This was intentional: it isolates the representational capacity of the operator. For example, fitting a Gaussian SVM on SHWSBic achieves 75.9% accuracy on DermaMNIST, beating all but one benchmark. However, the immediate linear separability provided by built‐in invariances was sought to be demonstrated, which would be obscured by an increasingly complex model.

**Table** **2** reports results on these benchmarks, which range from as few as 780 samples (BreastMNIST) to over 10 000 (DermaMNIST). Across all tasks, bispectral extensions provide highly discriminative features that were competitive with, and often surpass, deep learning baselines trained with far greater computational resources.

**Table 2:** Accuracy over MedMNIST benchmark datasets. We performed an initial hyperparameter sweep of H‐Net for each task over ring discretizations, number of filters, and angular frequencies. Other baselines are from refs. [39, 40].

Model	BreastMNIST	RetinaMNIST	DermaMNIST
ResNet‐18^[^ ^39^, ^40^ ^]^	86.3%	52.5%	73.5%
ResNet‐50^[^ ^39^, ^40^ ^]^	81.2%	52.8%	73.5%
auto‐sklearn^[^ ^39^, ^40^ ^]^	80.3%	51.5%	71.9%
AutoKeras^[^ ^39^, ^40^ ^]^	83.1%	50.3%	74.9%
Google AutoML^[^ ^39^, ^40^ ^]^	86.1%	53.1%	76.8%
H‐Net	84.4%	48.7%	72.4%
SHWS	84.6%	53.5%	72.1%
SHWB	78.2%	52.8%	69.7%
SHWBic	82.7%	54.5%	71.7%
SHWSB	85.9%	54.3%	71.0%
SHWSBic	86.5%	51.0%	72.2%

On BreastMNIST, SHWSBic outperforms all deep learning baselines. For the solid harmonic transform, ℓ = 9, *J* = 4 was used, and estimated the integrals using *L*_*p*_ norms for *p* ∈ {1, 2}. This task is strongly influenced by the global structure, demonstrating the data‐efficiency of the symmetry‐aware operators. They eliminate the need to learn such cues statistically from limited data, and thus excel. Further, the introduction of second‐order scattering coefficients yields improved results with SHWSBic over SHWBic, indicating that the co‐occurrence of strong symmetries between scales offers meaningful variability. Malignant tumors often exhibit hierarchical irregularities, which can be effectively modeled through the cross‐scale relationships encoded in scattering networks, and made richer by phase‐sensitive, nonlinear bispectral statistics.

Comparatively, RetinaMNIST depends heavily on localized, blot‐like structures. Here, SHWBic surpasses all baselines, while the addition of higher‐order scattering yields minimal gains. For the solid harmonic transform, ℓ = 7, *J* = 3 were used, and estimated the integrals using *L*_*p*_ norms for *p* ∈ 0.5, 1, 2. This suggests that the phase‐sensitive, multi‐scale encoding of localized symmetries alone provides a sufficiently discriminative representation for grading diabetic retinopathy.

Finally, DermaMNIST presents a more challenging setting, with seven classes discriminable by complex multiscale patterns. For the solid harmonic transform, ℓ = 8, *J* = 2 were used, and estimated the integrals using *L*_*p*_ norms for *p* ∈ 0.5, 1, 2, 4. With a comparatively larger training set, deep learning models retain an advantage due to their ability to learn symmetries directly from data, yet the operators remain competitive. It is worth emphasizing that the results were achieved with a single linear layer; while more expressive classifiers may close this gap, it is beyond the intentions of this work. Here, the multi‐scale wavelet decomposition captures fine‐to‐coarse structures, while scattering encodes inter‐scale dependencies that reflect relevant features such as border irregularity. Bispectral interactions enhance this by preserving phase relationships while encoding interactions between features at each scale.

While the Solid Harmonic Wavelet Bispectrum (SHWB) provides higher representational capacity through phase‐sensitive, third‐order correlations, simpler operators like SHWS can outperform SHWB in extremely low‐data regimes. This occurs because the additional complexity of bispectral interactions introduces variance when training data is very limited, whereas SHWS captures the dominant second‐order structure more robustly. As the dataset grows, SHWB's richer feature set consistently yields superior performance, demonstrating the benefits of higher‐order, phase‐sensitive encoding.

Overall, these benchmarks highlight how phase‐sensitive geometric invariances adapt to distinct diagnostic challenges: global symmetries in breast tumors, localized anomalies in retinal scans, and complex multiscale textures in dermascopic images. Across the operators, the same fundamental wavelets can be used to extract meaningful, domain‐relevant features without learning – rivaling the performance of deep learning in complex tasks with nonlinear decision boundaries. This demonstrates that embedding structural symmetries into the representation provides a powerful and data‐efficient alternative to deep learning, particularly in domains where data is scarce.

### 4.3 Astrophysics

A large area of astrophysics research centers on images of galaxies, which were governed by strong physical symmetries, and subject to transformations derived from natural forces: rotations and translations. This challenge is confounded by a key limitation in that only real galaxies can be sampled at one point in time, often from just one perspective. This gives rise to the use of simulated datasets in machine learning pipelines, which computationally follow natural evolutionary patterns of galaxies. While such simulations offer progressively larger quantities of data, they were costly and time‐consuming, meaning that deep learning will not always have sufficient data to learn complex, hierarchical symmetries statistically.

In particular, galaxy mergers exhibit complex morphological structures which evolve over time and historically have been challenging to identify with simpler statistical measures.^[^ ^41^, ^42^ ^]^ Deep learning is commonly used for simpler tasks, such as binary merger classification,^[^ ^43^ ^]^ where key symmetries can be learned from the data. However, regressing merger features requires the modeling of complex, continuous merger dynamics. This motivates the application of the operators which abstract nuisance transformations through built‐in symmetries and capture higher‐order, nonlinear interactions while preserving structural information through phase preservation – all without learning from data. Thus, a model trained on such features solely needs to learn variability within the simplified, compressed encoding. Both convex, linear models and non‐parametric methods (KNN regressors with inverse *L*_1_ distance weighting) were employed, which further enhance predictive capacity through the modeling of additional nonlinearities without a restrictive functional form.

A merger sample derived from IllustrisTNG^[^ ^44^ ^]^ was used with mass ratios μ ⩾ 1: 100 within 2.5 Gyrs of a merger for a redshift of up to 0.1, following the methodology in ref. [43] Data is extracted to a high spatial resolution, similar to space based data in the optical‐infrared, such as observed by the Hubble Space Telescope, which were cropped to 32 × the half stellar mass radius (as calculated from)^[^ ^44^ ^]^ and resize to 128 × 128. No observational noise was added. The source dataset is skewed due to the inherent properties of galaxy populations, making more major mergers rare. Thus, two distinct datasets were downsampled for both mass ratio, μ, and time since last merger coalescence, Δ*t*, by binning target values into 16 bins, each with a maximum of 2000 samples. This results in datasets of sizes 16,086 and 17,216 for μ and Δ*t*, respectively. For the solid harmonic transform, ℓ = 10, *J* = 6 were used, and estimated the integrals using *L*_*p*_ norms for *p* ∈ 0.5, 1, 2.

**Table** **3** details results on merger feature regression. With only a linear model, SHWS achieves a notable reduction over a random predictor, highlighting the importance of multi‐scale, structurally imposed symmetries. A further improvement is made with the non‐parametric KNN regressor, suggesting that additional nonlinearities can be flexibly exploited without reliance on a complex architecture. The bispectral operator (SHWSBic) offers the strongest results, consistently outperforming prior scattering techniques, and both Euclidean and non‐Euclidean deep learning baselines. These improvements underscore the importance of higher‐order interactions in capturing relative phase couplings between scales. Bispectral features encode how morphological structures captured by varying angular frequencies interact, where increasing the angular frequency captures progressively more granular directional patterns. Such representations were central to merger dynamics where features like tidal tails emerge from the interaction between global morphology and local perturbations.

**Table 3:** Results of merger feature regression on 128 × 128 resolution samples.

Model	Log μ		Δ*t* (Gyr)	
	MAE	RMSE	MAE	RMSE
Random predictor	0.6479	0.7932	1.2306	1.5065
Oriented scattering + linear	0.4119	0.4953	0.8025	0.9604
SHWS + linear	0.3876	0.4701	0.7781	0.9360
SHWS + KNN regressor	0.3539	0.4699	0.7264	0.9370
SHWSBic + KNN regressor	0.3322	0.4498	0.6633	0.9139
CNN	0.3564	0.4586	0.7206	0.9406
H‐Net	0.3581	0.4543	0.7345	0.9015

**Figure 3:**
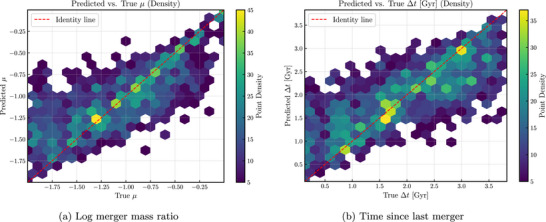
True‐predicted hexbin plots using K‐Nearest‐Neighbours regressor on Solid Harmonic Wavelet Scattering Bicoherence features. Hexbins with more than 5 samples are shown to highlight underlying trends and remove outliers.

**Figure** **3** illustrates the predictive power of the approach, with a clear positive correlation and uncertainty of ≈0.3 dex. This relationship reflects the ability of bispectral scattering representations to capture the nonlinear dynamics of galaxy mergers. Importantly, the smooth progression indicates that the representation goes beyond linear separability, preserving physically meaningful structure in which subtle evolutionary changes were reflected in the predicted values.

Together, these results reinforce the importance of embedding invariances and higher‐order statistics directly into representations, rather than relying on learned filters. By leveraging phase‐sensitive, multi‐scale interactions, the scattering bicoherence offers a data‐efficient alternative to deep learning.

### 4.4 Image Reconstruction

While the primary motivation of the Solid Harmonic Wavelet Bispectrum is to provide discriminative, symmetry‐aware, phase‐sensitive representations, it is equally informative to examine what information it precisely encodes about the underlying signal. Reconstruction tasks offer a natural avenue to explore this. In learned models, such as Generative Adversarial Networks,^[^ ^45^ ^]^ reconstructed image quality reflects how well the **learned** latent space captures the structure of data. In contrast, a similar approach was followed to^[^ ^46^ ^]^ on scattering‐based generative models: the Solid Harmonic Wavelet Scattering Bicoherence was evaluated as an encoder within an autoencoder architecture for image reconstruction, to observe what structural information is encoded **without learning**.

Further, an equivalent architecture was implemented using oriented Morlet wavelet scattering using Kymatio,^[^ ^34^ ^]^ which offers rotation‐covariant and locally translation‐invariant features, downsampled to preserve spatial structure. This approach was configured to produce a similar number of scattering coefficients to the bispectral approach. It was anticipated that the preservation of spatial structure through downsampling – as opposed to *L*_*p*_ pooling – will yield increasingly stable reconstructions. However, the phase preservation will retain key structural information, and so, offers an interesting.

Such architectures typically consist of two components: an **encoder** that maps an input image to a latent space (i.e., a compressed representation), and a decoder that reconstructs the image from this representation. The encoder can be written as a function *f*: **x**↦**z** where $x\in R^{H\times W\times C}$ denotes the input image and $z\in R^{d}$ is the latent space. Simultaneously, the decoder $g:z\mapsto \bar{x}$ aims to best reconstruct **x** from the crushed latent representation. The objective function is typically a reconstruction loss, minimized to drive the reconstructed image toward the input subject to desired properties such as image quality or perceptual similarity. Together, the autoencoder is therefore represented as $\bar{x}=g\left(f\left(x\right)\right)$, where the encoder learns to compress information into a meaningful latent representation.

In the approach, the encoder *f* was replaced with the operator ϕ_SHWSBic_: **x**↦**z** where **z** is the *L*_*p*_ pooled bispectral scattering coefficients, or similarly ϕ_Morlet_: **x**↦**z** where **z** is the downsampled Morlet scattering feature maps. For the solid harmonic transform, ℓ = 15, *J* = 4 were used, and estimated the integrals using *L*_*p*_ norms for *p* ∈ 0.5, 1, 2, 4; for Kymatio's implementation of the WST, *J* = 6 and *L* = 8 were used to obtain a comparable number of features.

The decoder follows a similar approach to Angles et al.^[^ ^46^ ^]^ Specifically, the latent space **z** is linearly projected to form a compact spatial feature map. From this initialization, a succession of transposed convolutional layers was employed, each doubling the spatial resolution while reducing the channel dimension, until reaching the target dimensions. Each layer is followed by ReLU activation, except for Sigmoid on the final layer to ensure pixel values in the same range as input images: [0, 1]. This differs slightly to the approach in ref. [46], which performed bilinear interpolations to upsample feature maps, before refining with convolutional layers. Transposed convolutions was opted because they directly integrate upsampling with learnable filtering.

This approach allows to directly probe the representational capacity of the encodings, highlighting what structural information they preserve in the absence of learned feature extraction. This was done by training on the UTKFace dataset,^[^ ^47^ ^]^ with a combined loss function that balances multiple objectives: mean squared error (MSE), *L*_1_ loss, and structural similarity (SSIM). Specifically, the loss function is defined: $L=0.5\;L_{\mathrm{MSE}}+0.2\;L_{1}+0.3\;L_{\mathrm{SSIM}}$. MSE captures pixel‐level fidelity, minimizing Euclidean distance. *L*_1_ loss encourages sparser, sharper reconstructions by penalizing absolute differences. Finally, structural similarity encourages the decoder to preserve local structures and textures – precisely the information the phase‐sensitive operator is designed to encode.

**Figure 4:**
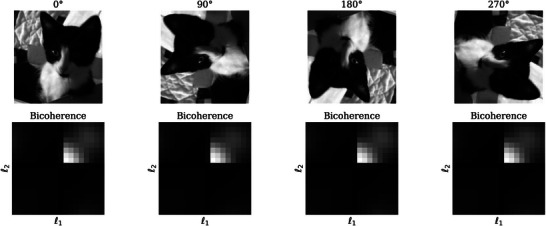
Solid harmonic wavelet bicoherence values for the same image sample from UTKFace^[^ ^47^ ^]^ rotated and mirrored.

**Figure 5:**
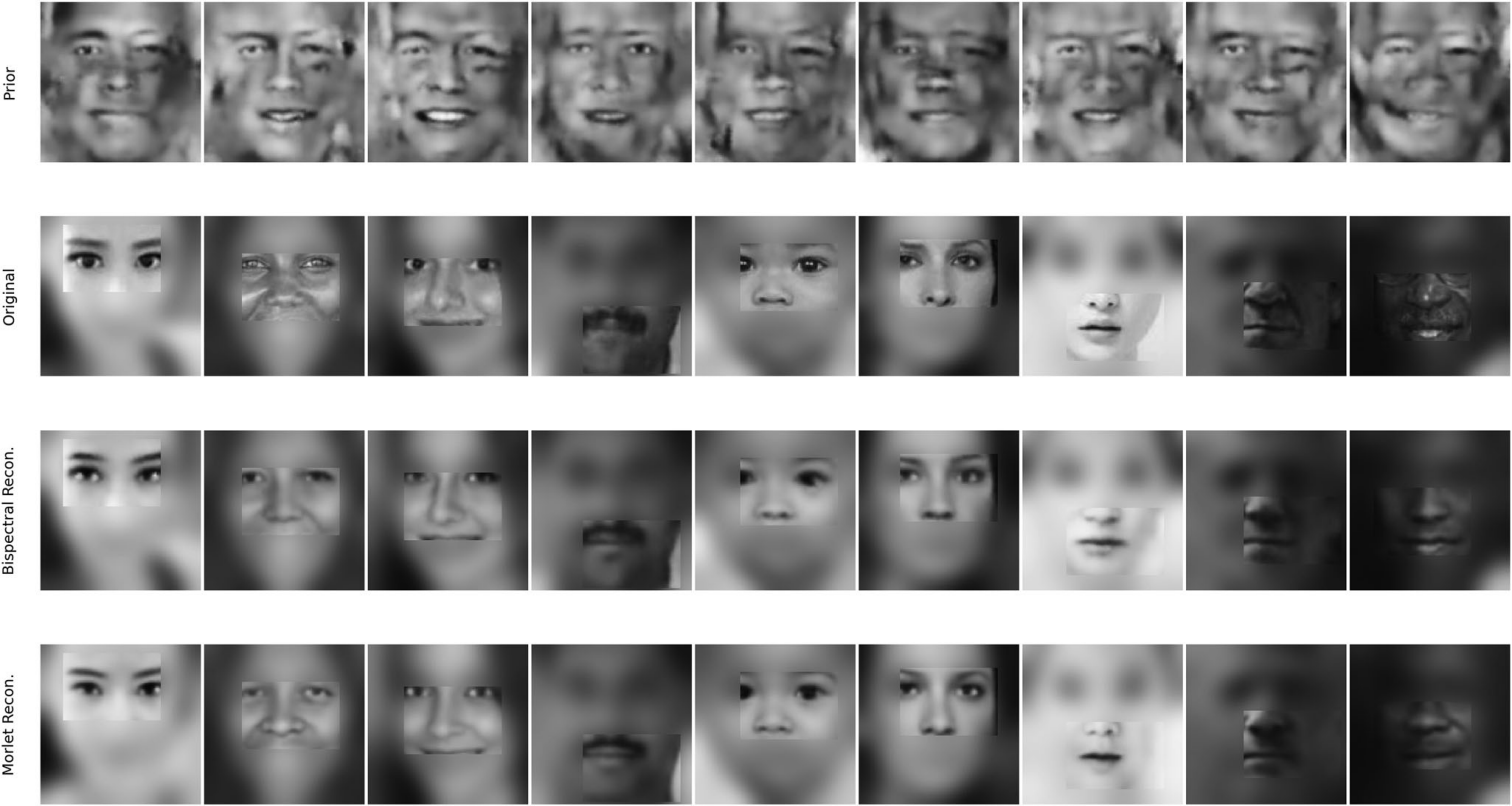
Comparison of image reconstructions. Top row: learned priors from a decoder trained on SHWSBic encodings (reconstructions from noise samples). Row 2: original images. Row 3: reconstructions from SHWSBic encodings. Row 4: reconstructions from Morlet scattering encodings. Images have been partially blurred to preserve subject privacy.

**Figure** **4** confirms that transformations such as mirroring and rotations reduce to identical Solid Harmonic Wavelet Scattering Bicoherence values, providing empirical evidence of rotational invariance. In reconstructions from noise, **Figure** **5** shows that the decoder has learned the broader geometry and key structural features of faces. This demonstrates that the SHWSBic encoding preserves crucial low frequencies capturing global shape, while also retaining higher‐frequency components necessary for localized facial features, enabling accurate reconstruction. These results further demonstrate that phase preservation contributes to maintaining critical structural information, even in the absence of a spatial structure within the latent space **z** – resulting from summation over spatial coordinates in *L*_*p*_ pooling. Comparing reconstructions from the latent spaces generated by the bispectral operator, and Morlet scattering, both evidently capture core facial structures. Importantly, the relative phase‐coupling of the scattering bicoherence over pooled coefficients enables reconstructions that were perceptually comparable to those obtained with downsampled Morlet scattering, despite the latter explicitly preserving spatial structure.

**Figure 6:**
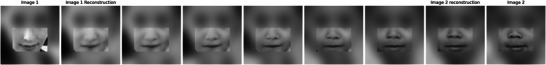
Reconstructions when sampling the latent space of scattering bicoherence encoding using linear interpolation. Images have been partially blurred to preserve subject privacy.

Moreover, a linear interpolation was performed over the latent space between two encoded images, **z**_1_ and **z**_2_. For an interpolation coefficient, α ∈ [0, 1], the interpolated latent space was sampled with: **z**_*interp*_ = (1 − α)**z**_1_ + α**z**_2_. **Figure** **6** shows the reconstructions over this interpolated latent space generated by ϕ_SHWSBic_. Decoding these interpolated vectors produces a smooth sequence of reconstructions, reminiscent of latent space interpolation with GANs.^[^ ^45^ ^]^ Notably, this structure emerges from the symmetry‐aware, higher‐order interactions of the scattering bicoherence, rather than learned feature extraction. Additionally, the gradual transition between encoded images demonstrates that the SHWSBic representation effectively encodes subtle variations in both global geometry and local features – as highlighted in the regression of merger features.

Overall, while bispectral reconstructions were marginally less stable than those from downsampled Morlet scattering, they preserve both structural and geometric features. To verify reconstruction quality, the average SSIM was calculated as 0.6717 ± 0.1626 for SHWSBic and 0.6539 ± 0.1021 for Kymatio‐based scattering. The Fréchet Inception Distance (FID) was 67.85 for the SHWSBic encoder and 59.72 for Kymatio. Interpreted alongside the qualitative characteristics in Figure 5, these results suggest that while SHWSBic does not capture the overall data distribution as effectively as Kymatio, it better preserves local statistics. The main insight is that a roto‐translation invariant algorithm like SHWSBic inherently loses more information than a locally translation‐invariant one like the standard Kymatio WST, particularly in datasets dominated by a single pose of the phase. However, the remaining information is sufficiently discriminative to reconstruct and describe the signal. This highlights that despite reducing features through *L*_*p*_ pooling, the symmetry‐aware, higher‐order interactions encode key structural information through relative phase coupling alone.

## 5 Conclusion

In this work, we provided an exposition of the Solid Harmonic Wavelet Bispectrum in two dimensions and its variants, demonstrating the crucial role that phase‐sensitive, higher‐order statistics play in building robust and data‐efficient representations. By structurally embedding roto‐translation invariance and encoding multi‐scale wavelet responses while preserving the relative phase across angular frequencies, our operators capture key structural content often discarded by conventional methods. The extension to the Solid Harmonic Wavelet Scattering Bispectrum enriches this by integrating cross‐scale dependencies. Our diverse set of benchmarks – from texture classification to regression of astrophysical features – demonstrate the importance of these mathematically imposed properties.

Our experiments consistently show that by explicitly encoding the coupling between wavelet responses, bispectral representations capture crucial structural information often lost in traditional scattering networks. In texture classification, the Solid Harmonic Wavelet Bispectrum shows marked improvements, particularly in low‐data regimes, underscoring not only the rich, discriminative features extracted without learning, but the importance of relative phase‐coupling. Over increasingly complex challenges in medical imaging, our bispectral operators proved competitive with, and often superior to, common deep learning architectures. This success, achieved with a single linear layer, stresses the importance of symmetry‐aware, geometric invariances by design, which simplify the subsequent decision boundary by abstracting redundant transformations. These findings were solidified in the regression of galaxy merger features, where the Solid Harmonic Wavelet Scattering Bicoherence outperformed both conventional scattering and deep learning baselines. The ability to model nonlinear merger dynamics of features like tidal tails, which arise from the interaction of structures at different scales and orientations, is a result of the multi‐scale, phase‐sensitive encoding which is increasingly data‐efficient due to built‐in geometric invariances. Finally, image reconstruction experiments reveal that despite the summation of spatial information via *L*_*p*_ pooling, the relative phase‐coupling alone retains sufficient structural detail for high‐quality reconstruction and meaningful latent space interpolation.

While deep learning models may still hold an advantage on very large datasets where increasingly complex, nonlinear decision boundaries can be learned, our findings demonstrate that symmetry‐aware wavelet‐based operators excel in data‐scarce domains governed by natural symmetries. Future work could explore hybrid architectures that leverage the rich, handcrafted features of the Solid Harmonic Wavelet Bispectrum as input to neural networks – combining the data efficiency of structurally imposed geometric invariances with the flexible capacity of learned models.

To conclude, the Solid Harmonic Wavelet Bispectrum offers a principled framework for signal analysis: moving beyond second‐order statistics allows the modeling of phase‐sensitive, higher‐order interactions proven to be increasingly informative across scientific disciplines.

## Conflict of Interest

The authors declare no conflict of interest.

## Data Availability

The data that support the findings of this study are available from the corresponding author upon reasonable request.
